# Internet-based eHealth technology for emotional well-being among the older adults with a family cancer history: full mediation effects of health information self-efficacy and cancer fatalism

**DOI:** 10.1186/s40359-024-01701-0

**Published:** 2024-04-25

**Authors:** Yuyuan Kylie Lai, Jizhou Francis Ye, Qiawen Ran, Harris Song Ao

**Affiliations:** 1 Institute of Collaborative Innovation, University of Macau, Macao, China; 2 Department of Communication, University of Macau, Macao, China; 3 China Petroleum Pipeline Engineering CO., LTD. International, Hebei, China; 4 University of Macau Avenida da Universidade, Room 1001, N21, Research Building, Taipa, Macao

**Keywords:** eHealth, Family cancer history, Health information self-efficacy, Cancer fatalism, Emotional well-being

## Abstract

**Background:**

Older adults with a family cancer history (FCH) face an increased cancer risk, which may adversely impact their emotional well-being. Internet-based eHealth technologies (IETs) provide a potential solution to this challenge. This study examines the influence of using IETs on the emotional well-being of older adults with FCH. It also delves into the mediating pathways through health information self-efficacy and cancer fatalism.

**Methods:**

This study conducted a mediation analysis using data from the Health Information National Trends Survey (HINTS 6) collected from March 2022 to November 2022, focusing on older adults with FCH who had previously searched for cancer-related information (*N* = 1,280).

**Results:**

In the mediation model, no positive direct associations between IETs usage and emotional well-being were found. Only health information self-efficacy and cancer fatalism were found to mediate the relationship between IETs usage and emotional well-being serially (β = 0.007, 95% CI [0.003, 0.012]).

**Conclusions:**

The findings inform health information professionals and healthcare practitioners on enhancing the impact of IETs usage on individual health information self-efficacy, which mitigates cancer fatalism, contributing to better emotional well-being in the digital era.

## Background

In both clinical and public health settings, gathering a family cancer history (FCH) serves as a cost-effective, straightforward approach for assessing cancer risk and informing preventative measures [1]. Notably, a heightened awareness of FCH can pose significant emotional risks [2]. For instance, having a family member with cancer will increase one’s perceived risk of inheriting a genetic predisposition to the disease, leading to heightened worry and anxiety. Witnessing a family member’s unsuccessful treatment for advanced cancer may reinforce one’s fatalistic belief that cancer is a hopeless and incurable disease [3]. Consequently, patients with FCH are prone to experience elevated levels of anxiety, distress, and depression [4]. This is particularly true for older populations, who confront not only the uncertainty of prospective cancer development but also the challenges of aging [5, 6].

Effectively improving the emotional well-being of older populations with FCH requires the efficient use of healthcare information technologies, including Internet-based electronic health (eHealth) technologies. The World Health Organization [7] defines eHealth as the cost-effective and secure utilization of information and communications technologies to aid health and health-related fields, such as health surveillance and healthcare services. Thanks to growing digital technologies, 75% of adults above 65 in the United States (U.S.) have become Internet users in 2021 [8]. Telehealth visits among U.S. older adults have risen from 4% in 2019 to 30% in 2020 [9]. In this context, the term “Internet-based eHealth technologies (IETs)” encompasses a broad range of tools, referring to the utilization of the Internet to facilitate patient interactions with healthcare providers or others for remote forms of preventive, curative, and recovery-oriented care [10]. According to the Centers for Disease Control and Prevention [11], IETs can perform various functions, such as allowing patients to communicate with their healthcare providers via the web and searching for online information regarding nutrition, weight, and fitness. Empirical evidence suggested that IETs were useful resources for patient self-care, offering channels for looking up test results, scheduling appointments with healthcare providers, and chatting with professionals [12, 13]. This can empower individuals by providing equal access to essential healthcare resources, services, and information (e.g., medical results), and fostering active participation in healthcare processes for both patients and providers [12, 14]. Previous research has shown that IETs can help enhance patients’ sense of companionship, social support, life satisfaction, and patient activation [15, 16], all of which are crucial for their emotional well-being.

Although the positive association between IETs usage and health outcomes has been documented in some studies [14, 15], little is known about how IETs usage by older adults with FCH is associated with their emotional well-being and the underpinning mediation chain. Older adults with FCH face a unique vulnerability to emotional distress; on the one hand, empirical evidence has suggested a positive correlation between FCH and psychological distress, even controlling demographic variables [17]. On the other hand, aging is associated with an increased incidence of most cancers, particularly after midlife [5]. The American Cancer Society [18] estimated that 74% of people diagnosed with cancer in the U.S. will be age 65 or older by 2040. This may exacerbate the perceived cancer threat of older patients with FCH and induce feelings of helplessness and hopelessness. Therefore, addressing this population’s emotional health needs is critical. As illustrated by previous studies [19, 20], later-life depression is often accompanied by older adults’ chronic diseases, cognitive and physical impairments, and psychosocial challenges, such as loneliness. Building on the aforementioned rationales, the present study focused on the emotional well-being of older adults with FCH and explored the mediating mechanism of how IETs usages enhance emotional well-being.

The eHealth Enhanced Chronic Care Model (eCCM) [21] provides a sound theoretical framework for our investigation. The model proposes that eHealth tools can facilitate individual health outcomes by performing five main functions: (1) self-management support, (2) delivery system design, (3) clinical decision support, (4) clinical information systems, and (5) eHealth education. Scholars and practitioners widely use the eCCM to design and evaluate IETs for patients. Within this framework, health information self-efficacy (i.e., the belief that one can access and effectively use health information when needed) [22] is considered a crucial intermediate variable that stems from the concept of health self-efficacy. Relatedly, health self-efficacy pertains to people’s belief in their capabilities in successfully performing behaviors to achieve health-related goals [23, 24]. Previous research has demonstrated that personal health self-efficacy mediates eHealth use and health outcomes, such as psychological health [25] and quality of life [26]. In the online continuum where health-related information is plentiful and widely disseminated, we contend that health information self-efficacy possibly plays a particularly salient role in delving into the relationship between IETs usage and emotional well-being. Nonetheless, nascent research pays attention to the particular threat to older patients with FCH, especially their potential susceptibility to cancer fatalism - a personal belief that cancer is predetermined or inevitably fatal [27]. To contribute to this underexplored area, we indicate that cancer fatalism may be another crucial psychological factor influencing the relationship between IETs usage and emotional well-being. Specifically, by providing decisional support and patient education, IETs usage can enhance patients’ capability and confidence to seek and utilize cancer-related information (i.e., health information self-efficacy), which helps reduce their cancer fatalistic beliefs, leading to enhanced emotional well-being.

Individual health information self-efficacy is particularly substantial in the online clinical setting, where patients may encounter a plethora of ambiguous, uncertain, and complex information that can induce information overload and negative affective responses [28]. By using IETs, patients can access more credible and up-to-date information for self-health administration and eHealth skill enhancement. This can boost their confidence in acquiring, processing, and employing health information. Especially for our study population, scholars have documented that lower confidence in self-care is often associated with aging [6] and FCH [29], thus necessitating a solution to improve their confidence to seek out resources for good health. Several studies have substantiated the direct link between IETs usage and health information self-efficacy. A national survey in cancer care found that more frequent use of patient portals was statistically related to higher health information self-efficacy [30]. A systematic review suggested that using digital care platforms could augment patients’ knowledge of cancer, their sense of control, and their perceived ability to access and employ cancer-related information [31].

Furthermore, health information self-efficacy may play a facilitative role in reducing patients’ cancer fatalism. Cancer fatalism denotes one’s belief that cancer is predetermined or inevitably fatal [27]. This belief can influence individuals’ health behavior regarding cancer detection. Understandably, patients’ fatalistic beliefs will be diminished as they become confident in finding, evaluating, and applying information to address health problems. A U.S. longitudinal study demonstrated that patients who perceived themselves as capable of seeking, understanding, and evaluating eHealth information and applying the knowledge gained to address health issues were less likely to view cancer as unpreventable [32]. Similarly, a recent study examining the association between social media use and emotional health found a notable negative association between people’s perception of their capacity to implement their health-related goals successfully and their fatalistic beliefs [33].

Individual health information self-efficacy can be a driving force of emotional well-being, as cancer research has repeatedly verified the perceived capability in self-health management as a key determinant of patients’ emotional well-being [6, 34]. Theoretical foundations for this line of research are provided by several theories, such as the self-determination theory [35] and the three-stage model of health promotion [36], which posit health information self-efficacy as an intrinsic motivation that drives people to pursue health [37]. In contrast, cancer fatalism has long been considered a detrimental barrier to patients’ emotional well-being. On the one hand, cancer fatalism can trigger fear of cancer occurrence, which can cause depressive and anxiety symptoms [38]. On the other hand, cancer fatalism can deter patients from accessing quality healthcare as they believe that diagnosis or treatment is inefficient and that having cancer signifies imminent death, thereby imposing a heavy burden on their mental health. Supporting this notion, a systematic review involving 1,281 patients indicated that patients’ cancer fatalism was tied to greater psychological distress [39].

Based on the discussion above, we propose that health information self-efficacy and cancer fatalism constitute a serial mediating mechanism that connects the first construct and the final one. This route was partially by an earlier study that conceptualized health information self-efficacy and fatalistic beliefs as co-occurring mediators that account for how Internet health information seeking improves cancer-related health outcomes [40]. The additional theoretical justification offered by the three-stage model of health promotion [36] contends that technology use seldom directly leads to desirable cancer-related outcomes. Instead, the effect is likely to be mediated by a range of intermediate outcomes, such as self-efficacy in consuming eHealth contents, and attitude change, which corresponds to cancer fatalism in our study. Thus, based on empirical evidence and theoretical foundations, the following direct and indirect relationships between IETs usage and emotional well-being were put forward:

**H1**: IETs usage is positively associated with emotional well-being (direct effect).

**H2**: Health information self-efficacy mediates the relationship between IETs usage and emotional well-being (indirect effect).

**H3**: Cancer fatalism mediates the relationship between IETs usage and emotional well-being (indirect effect).

**H4**: Health information self-efficacy and cancer fatalism sequentially mediate the relationship between IETs usage and emotional well-being (indirect effect).

## Methods

### Sample

The current study utilized secondary data from the Health Information National Trends Survey (HINTS), which was collected from March 7 to November 8, 2022 (HINTS 6). HINTS is a nationally representative survey to assess American citizens’ health information behaviors and outcomes [41]. The HINTS 6 survey employed a two-stage stratified sampling method to collect data from a representative sample of U.S. adults [42]. The first stage involved selecting a random sample of residential addresses from a comprehensive file, while the second stage was to choose one adult from each household in the sample [42]. Based on minority status and geographic location, the HINTS 6 sample was divided into four strata: high minority urban, high minority rural, low minority urban, and low minority rural [42]. This stratification aimed to increase the representation of respondents from rural and high-minority areas in the sample. A total of 6,252 participants completed the survey (response rate = 28.07%). As the current study focused on older adults with FCH who have cancer information-seeking experience, the respondents were first filtered based on an age criterion of not less than 55 years old (*N* = 3,494). Further, we identified those with first- or second-degree biological relatives who had been diagnosed with cancer and those who had looked for cancer information based on two dichotomized questions. Therefore, the final sample included 1,280 participants. This study used secondary data. The HINTS data meet ethics standards and have obtained ethics approval. Additionally, ethical approval was not required for this study since the HINTS data we use is publicly available.

### Measurement

*IETs usage* was measured with four items unique in HINTS, drawn from previous research [12, 43]. Respondents were asked in the past 12 months, whether they had used the Internet to (1) look for health or medical information, (2) send a message to a health care provider or health care providers’ office, (3) view medical test results, (4) make an appointment with a health care provider. Responses were dichotomous (0 = “*no”*, 1 = “*yes”*) and added up to create a composite scale (Cronbach’s alphas = 0.71).

*Health information self-efficacy* was measured using one single item adapted from previous studies [44, 45]. Participants were asked to indicate how confident they were in finding helpful health resources on the Internet. Responses were scored on a 5-point Likert scale ranging from “1 = *not confident at all*” to “5 = *completely confident*”.

*Cancer fatalism* was measured through a four-item scale originally derived from HINTS, widely used in a spectrum of studies [46–48]. These questions ask respondents how much they agree: (1) “It seems like everything causes cancer,” (2) “There’s not much you can do to lower your chances of getting cancer,” (3) “There are so many different recommendations about preventing cancer, it’s hard to know which ones to follow,” (4) “When I think about cancer, I automatically think about death.” A 4-point scale was employed, ranging from 1 = “*strongly agree”* to 4 = “*strongly disagree”*. Responses were reversely scored, with higher scores representing higher levels of fatalistic beliefs about cancer (Cronbach’s alphas = 0.65).

Prior research indicated that these four items separately evaluated the fatalistic belief about cancer causes, the fatalistic belief about cancer prevention, perceived ambiguity about cancer prevention recommendations, and the fatalistic belief of cancer consequences [47, 49]. This aligns with the multifaceted construct of cancer fatalism, where confusion and uncertainty about cancer are indicative of the fatalistic beliefs of powerlessness over cancer incidence. Although the cancer fatalism scale exhibits low reliability, this aligns with previous research that employed similar measures. Thus, we chose to adopt the four-item scale to represent cancer fatalism.

*Emotional well-being* was measured through the Patient Health Questionnaire 4 (PHQ–4) [6, 50], which asked participants over the past 2 weeks how often they have been bothered by: (1) little interest or pleasure in doing things, (2) feeling down, depressed or hopeless, (3) feeling nervous, anxious or on edge, (4) not being able to stop or control worrying. All items were rated on a 4-point scale ranging from 1 = *nearly every day* to 4 = *not at all* (Cronbach’s alphas = 0.86).

To ensure accuracy in our study, we included control variables such as socio-demographic and health-related variables. Control variables include age, gender, ethnicity, household income, education, health insurance, and general health status. Previous research has indicated that emotional well-being may vary across populations with distinct demographic backgrounds [51, 52] and health status [53, 54]. Therefore, we controlled for demographics, including age, gender, ethnicity, household income, education, and health insurance, as well as general health status (five levels) in the current study. Age was treated as a continuous variable. Gender was categorized as male and female based on self-reported gender listed on the individual’s birth certificate. Ethnicity was categorized into five groups (1= “*Non-Hispanic White*”, 2= “*Black or African American*”, 3= “*Hispanic*”, 4= “*Asian*”, 5= “*Other*”). Household income was categorized into five groups (from 1 = “*less than $20,000”* to 5 = “*$75,000 or more”*). Education was categorized into four groups (from 1 = “*less than high school”* to 4 = “*college graduate or more”*). Health insurance is dichotomous coded (1 = “*yes*”, 0 = “no”).

### Statistical analysis

Data analysis was conducted using SPSS 26. First, we utilized descriptive statistics to depict demographic characteristics. Second, we assessed partial correlations between all study constructs. Then, the proposed mediation relationships were tested using linear regression and were examined using Model 6 from the SPSS macro PROCESS [55]. Regarding the indirect effect, we applied 10,000 bootstrap samples to estimate the 95% bias-corrected confidence intervals (CIs). Potential confounding variables were controlled for in all models and standardized coefficients were displayed. The level for statistical significance was set at alphas = 0.05.

## Results

### Descriptive analyses

The mean age was around 68 (SD = 8.21). The female participants (64.5%) were more than the male ones (35.5%). The majority were Non-Hispanic White (73.2%). Many of the participants had health insurance (97.8%), had received a college education or above (53.6%), had an average annual household income of more than U.S. $50,000 (62.5%), and had a medium level of general health status (M = 3.36, SD = 0.93). The detailed demographic information is summarized in Table 1.

**Table 1:** Demographic of the study sample (*N* = 1,280)

Variables	Value
Gender (n. %)	
Male	454 (35.5)
Female	824 (64.5)
Ethnicity (n. %)	
Non-Hispanic White	892 (73.2)
Black or African American	151 (12.4)
Hispanic	119 (9.8)
Asian	25 (2.1)
Other	31 (2.5)
Education (n. %)	
Less than high school	43 (3.4)
High school graduate	166 (13.0)
Some college	384 (30.0)
College graduate or more	685 (53.6)
Household income (n. %)	
Less than $10,000	42 (3.5)
$10,000 to < $15,000	39 (3.3)
$15,000 to < $20,000	50 (4.2)
$20,000 to < $35,000	155 (13.0)
$35,000 to < $50,000	163 (13.6)
$50,000 to < $75,000	233 (19.5)
$75,000 to < $100,000	183 (15.3)
$100,000 to < $200,000	223 (18.7)
$200,000 or more	107 (9.0)
Health insurance (n. %)	
Yes	1,246 (97.8)
No	28 (2.2)
General health status (five levels, Mean $$ \pm $$SD)	3.36 $$ \pm $$0.93
Age (years, Mean $$ \pm $$SD)	68.09 $$ \pm $$8.21

### Relationships among IETs usage, health information self-efficacy, cancer fatalism, and emotional well-being

Table 2 shows partial correlations among study variables controlling for demographic variables. The correlations between IETs usage and health information self-efficacy (*r* =.194, *p* <.001) were significantly positive. Meanwhile, cancer fatalism was negatively associated with emotional well-being (*r* = −.146, *p* <.001), IETs usage (*r* = −.087, *p* =.004), and health information self-efficacy (*r* = −.151, *p* <.001).

**Table 2:** Partial correlations among study variables

	α	Mean $\pm$SD	1	2	3	4
1. Emotional well-being	0.86	3.56 $\pm$0.61	–			
2. IETs usage	0.71	3.08 $\pm$1.14	0.010	–		
3. Health information self-efficacy	–	3.51 $\pm$0.81	0.002	0.194***	–	
4. Cancer fatalism	0.65	2.31 $\pm$0.59	–0.146***	–0.087**	–0.151***	–

*Note*. *: *p* <.05; **: *p* <.01; ***: *p* <.001. IETs: Internet-based eHealth technologies; Numbers are Pearson correlation coefficients; Covariates: gender, age, ethnicity, education, health insurance, household income, and general health status.

**Fig. 1:**
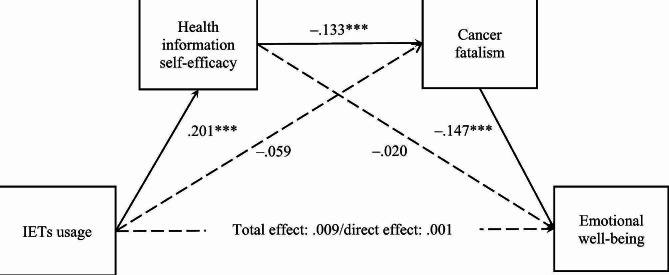
Results of the Conceptual Framework

**H1** posited the positive direct association between IETs usage and emotional well-being. As illustrated in Table 3; Fig. 1, IETs usage was not associated with emotional well-being (β = 0.001, 95% CI [− 0.031, 0.032]), so **H1** was not supported. **H2** and **H3** predicted the separate mediating roles of health information self-efficacy and cancer fatalism between the relationship between IETs usage and emotional well-being. However, neither of these indirect effects pass the statistical threshold (95% CI contained zero) for health information self-efficacy (β = −0.004, 95% CI [− 0.017, 0.009]) or cancer fatalism (β = 0.009, 95% CI [− 0.001, 0.020]). Therefore, **H2** and **H3** were not supported. **H4** proposed that IETs usage was associated with emotional well-being through health information self-efficacy and cancer fatalism in sequence. The indirect effect was statistically significant (β = 0.004, 95% CI [0.002, 0.007]), thereby supporting **H4**.

**Table 3:** Mediation analysis

	Outcomes										
Predictors	Health information self-efficacy			Cancer fatalism			Emotional well-being			Emotional well-being	
	β	SE		β	SE		β	SE		β	SE
***Main predictors***											
IETs usage	0.201***	0.022		–0.059	0.016		0.001	0.016		0.009	0.016
Health information self-efficacy				–0.133***	0.021		–0.020	0.022			
Cancer fatalism							–0.147***	0.031			
*R* ^2^	0.094			0.165			0.185			0.167	
*F*	5.659***			10.237***			11.215***			10.914***	
Sociodemographic and health-related controls	√			√			√			√	
***Standardized indirect effects***							Coefficient	Boot SE		Boot LLCL	Boot ULCL
Mediator: Health information self-efficacy							–0.004	0.006		–0.017	0.009
Mediator: Cancer fatalism							0.009	0.005		–0.001	0.020
Mediators: Health information self-efficacy & cancer fatalism							**0.004**	**0.001**		**0.002**	**0.007**

*Note*. *: *p* <.05; **: *p* <.01; ***: *p* <.001. IET: Internet-based eHealth technology; LL: lower limit; CI: confidence interval; UL: upper limit; SE: standard error. Standardized betas are shown in each cell. Bootstrap sample size equal to 10,000.

### Comparisons by age cohorts 

An independent sample *t*-test was conducted to investigate differences in IETs usage and psychological outcomes between older adults seeking cancer-related information and those who do not, both of whom have FCH. As illustrated in Table 4, compared to elderly non-seekers with FCH, elderly seekers of cancer information with FCH reported a significantly higher score in terms of Internet-based health service usage (M_*seeker*_ = 3.08, M_*non−seeker*_ = 2.26, M_*diff*_ = 0.82, Cohen’s *d* = 0.64, *p* <.001) and information self-efficacy (M_*seeker*_ = 3.40, M_*non−seeker*_ = 2.99, M_*diff*_ = 0.41, Cohen’s *d* = 0.43, *p* <.001), as well as a lower level of cancer fatalism (M_*seeker*_ = 2.35, M_*non−seeker*_ = 2.56, M_*diff*_ = − 0.21, Cohen’s *d* = 0.31, *p* <.001). Although emotional well-being did not show significant differences (M_*seeker*_ = 3.52, M_*non−seeker*_ = 3.54, M_*diff*_ = − 0.02, Cohen’s *d* = 0.03, *p* =.43), the results suggest a division in IET usage between seekers and non-seekers of cancer information, which potentially leads to variation in health information self-efficacy and cancer fatalism outcomes.

**Table 4:** Mean differences of focal variables between elderly individuals with FCH engaged in health information seeking and non-seeking

	Elderly seekers of health information with FCH (Mean $\pm$SD)	Elderly non-seekers of health information with FCH (Mean $\pm$SD)	Cohen’s d
Emotional well-being	3.52 $\pm$ 0.65	3.54 $\pm$ 0.68	0.03
IET usage ***	3.08 $\pm$ 1.14	2.26 $\pm$ 1.41	0.64
Health information self-efficacy***	3.40 $\pm$ 0.90	2.99 $\pm$ 1.01	0.43
Cancer fatalism***	2.35 $\pm$ 0.62	2.56 $\pm$ 0.68	0.31

*Note*. ***: *p* <.001. FCH: family cancer history; IET: Internet-based eHealth technology.

## Discussion

The current study breaks new ground to explore the effect of IETs usage on the emotional well-being of older adults with FCH. Our evidence indicates a *full mediation* [56], suggesting that the positive effect of IETs usage on emotional well-being is contingent upon the serial chain of health information self-efficacy and cancer fatalism. This empirically validates the central proposition of the eCCM [21], which posits that health information self-efficacy is a vital consequence of eHealth use and can facilitate individual skills and knowledge in self-management, resulting in improved health outcomes. Also, the negative association between health information self-efficacy and cancer fatalism is congruent with previous evidence showing that older adults with higher response efficacy and self-efficacy beliefs tended to report lower levels of cancer fatalism [57]. Such finding is particularly prominent for older patients with FCH who often encounter higher cancer fatalism and emotional stress [58] but lack adequate access to face-to-face healthcare to tackle these issues due to age-related physiological declines. Compared to traditional offline healthcare systems, IETs offer patients more convenience, customization, and involvement in their care [12], which can foster more self-confidence and social connectedness among older adults with FCH. As such, IETs can serve as complementary or supplementary avenues for them to gain psychological empowerment and reduce cancer fatalism, which plays a pivotal role in fortifying their optimistic health beliefs, improving self-care intention, and promoting wellness.

However, the direct relationship between IETs usage and emotional well-being is insignificant, suggesting that merely utilizing IETs may not confer immediate benefits to older adults. This result is aligned with previous findings suggesting that health-related Internet use does not directly enhance patients’ health outcomes (e.g., emotional well-being and lifestyle change) but rather through psychological mechanisms such as patient activation and empowerment [14, 59]. One plausible explanation is that the adoption of IETs may have negative psychological outcomes, such as cancer worry [60], which can offsite the positive impact of IETs usage. When IETs are still in their infancy, problems with inaccurate or inconsistent information exchanged via eHealth tools are common. Exposure to conflicting and ambiguous cancer-related information in the cancer care arena could result in increased cancer-risk perceptions and worry, which may adversely affect emotional health [49]. Moreover, such findings can also be interpreted through the lens of the eCCM, positing that IETs usage could lead to better health, but only when patients have sufficient health literacy to comprehend and apply eHealth content for self-care [21]. Different from the concept of health information self-efficacy, eHealth literacy captures one’s actual ability to search, comprehend, evaluate, and apply health information from electronic sources to address health issues rather than their perceived capacity [61]. Although scholarship has hypothesized a positive link between eHealth literacy and self-efficacy in the information [62, 63], people who are confident in managing health information may not have excellent skills in digesting substantial health-related information, especially via online channels. Unfortunately, older adults in the U.S. consistently show poor eHealth literacy [64], which hinders their ability to utilize the potential of IETs fully. Therefore, patient education for the elderly to enhance health literacy is a prerequisite for facilitating their effective usage of IETs. Another possible reason for the null finding may be the privacy concern in online environments. The proponents of eCCM have indicated that some patients were worried about the lack of control and autonomy over their health data when using IETs [21]. This may hinder them from disclosing personal symptoms and emotions online, reducing their chances of receiving empathy and reassurance from others. This argument is in line with the notion of self-determination theory, which suggests that autonomy is one of the intrinsic motivations that drives individuals to pursue mental health and well-being [65]. Therefore, it is imperative to ensure patient privacy and educate patients on how to use IETs effectively before they can reap sufficient benefits to escalate emotional well-being.

### Clinical implications

The findings of this study can offer significant practical implications for the improvement of emotional well-being. We first demonstrate the feasibility and effectiveness of eHealth tools for improving emotional well-being among older adults. However, these tools require adaptation to the specific needs and preferences of the older population, considering their possible limitations in vision, hearing, and memory [66]. For instance, clear visual displays and user-friendly audio feedback can facilitate older adults’ adoption and engagement of eHealth technologies, resulting in a virtuous interactive experience. Second, we ascertain health information self-efficacy and cancer fatalism as pivotal mediators in the relationship between Internet health information seeking and health outcomes. Consequently, health information professionals should endeavor to augment information literacy and health literacy among older adults, empowering them to critically screen, evaluate, and utilize online health information. Furthermore, to avert the exacerbation of cancer fatalism among individuals with FCH, regulation from government agencies and health institutions is essential to curb the spread of misinformation about the unpreventability and inevitability of cancer. IETs usage can also affect the younger population’s cognitive and affective outcomes. A systematic review found that eHealth tools could assist younger adults in establishing a connection with their healthcare providers and tracking their mood, stress levels, and daily activities, leading to enhanced mental health outcomes [67]. Young adults with FCH, particularly those possessing great accessibility and proficiency with technology, may experience substantial benefits by incorporating IETs into their routines.

### Study limitations

This study has several limitations that warrant further research. First, the cross-sectional design of this study prevents us from establishing a causal relationship between the usage of IETs and emotional well-being. Moreover, it is plausible that the emotional well-being of individuals may vary depending on their health status, including different stages of a disease. Although our study has taken into account general health status as a covariate, future research could adopt a longitudinal survey or experimental design further to explore the relationship between IETs adoption and emotional well-being. Second, with the advancement of health-related technologies, the current measure of IETs usage used in the study may not fully capture all the functionalities that this tool can provide. Hence, future research could incorporate the most up-to-date functions of IETs to better assess their impact on emotional well-being. Third, health information self-efficacy was assessed using a single item, which might cause bias. Also, the measure used in this study to identify elderly seekers of cancer information was not specific to online channels. Future studies should include multiple items to measure health information self-efficacy and measures of concrete channels for cancer information seeking.

## Conclusion

As the aging population faces increasing challenges in cancer care, it is crucial to understand how communication and technology can enhance their emotional well-being. In light of this, this study examines the impact of IETs usage on emotional well-being among older adults with FCH. Our results suggest that the influence is likely to be indirect through the mediating roles of health information self-efficacy and cancer fatalism. Findings have implications for information professionals and healthcare organizations to create a credible and supportive online environment, deliver quality eHealth information, and improve patient literacy, ultimately improving patients’ emotional well-being.

## Data Availability

The HINS data can be found at https://hints.cancer.gov/.

## Abbreviations

IETs: Internet-based eHealth technologies
FCH: Family cancer history
eCCM: eHealth enhanced chronic care model
